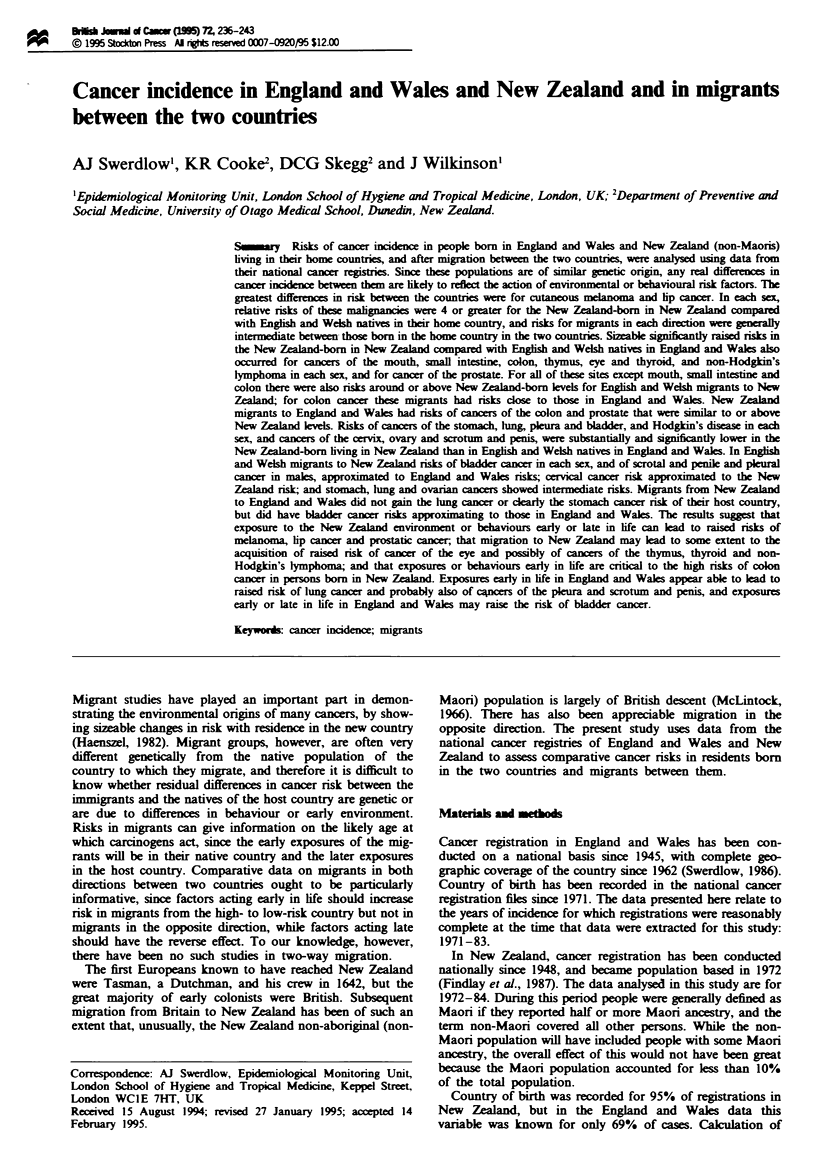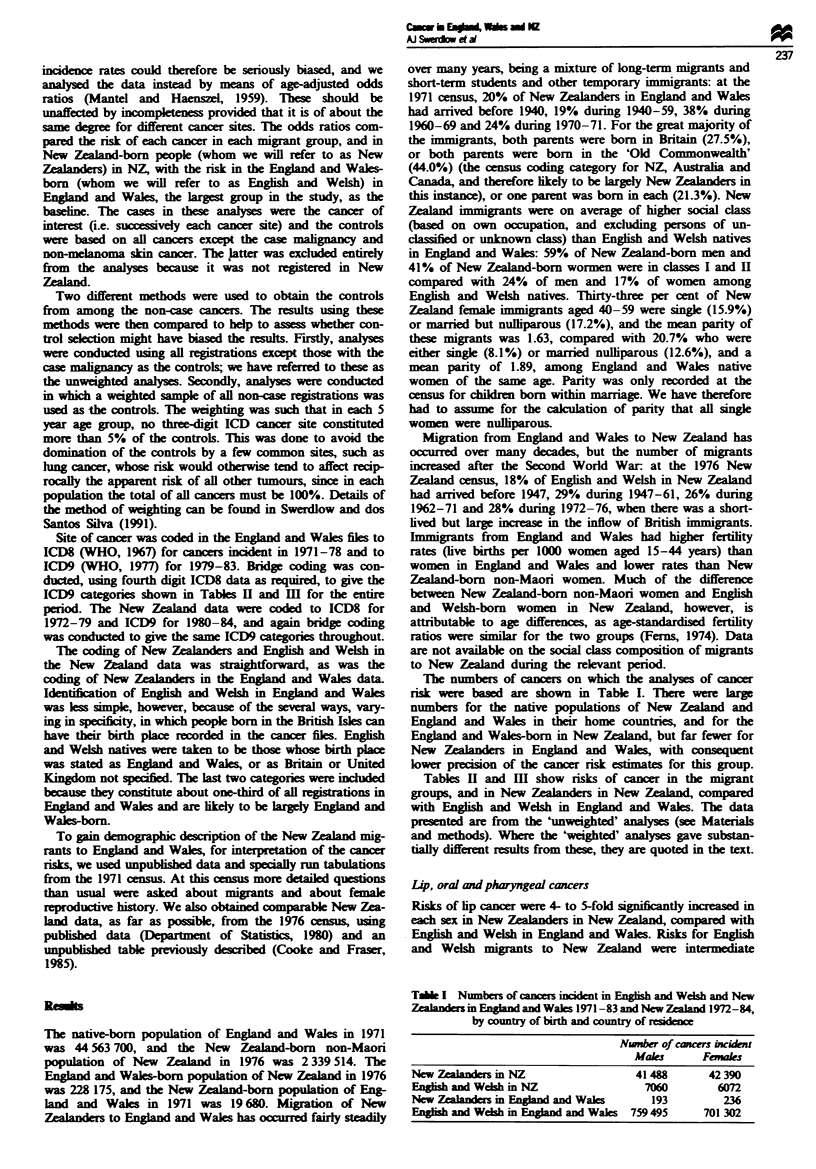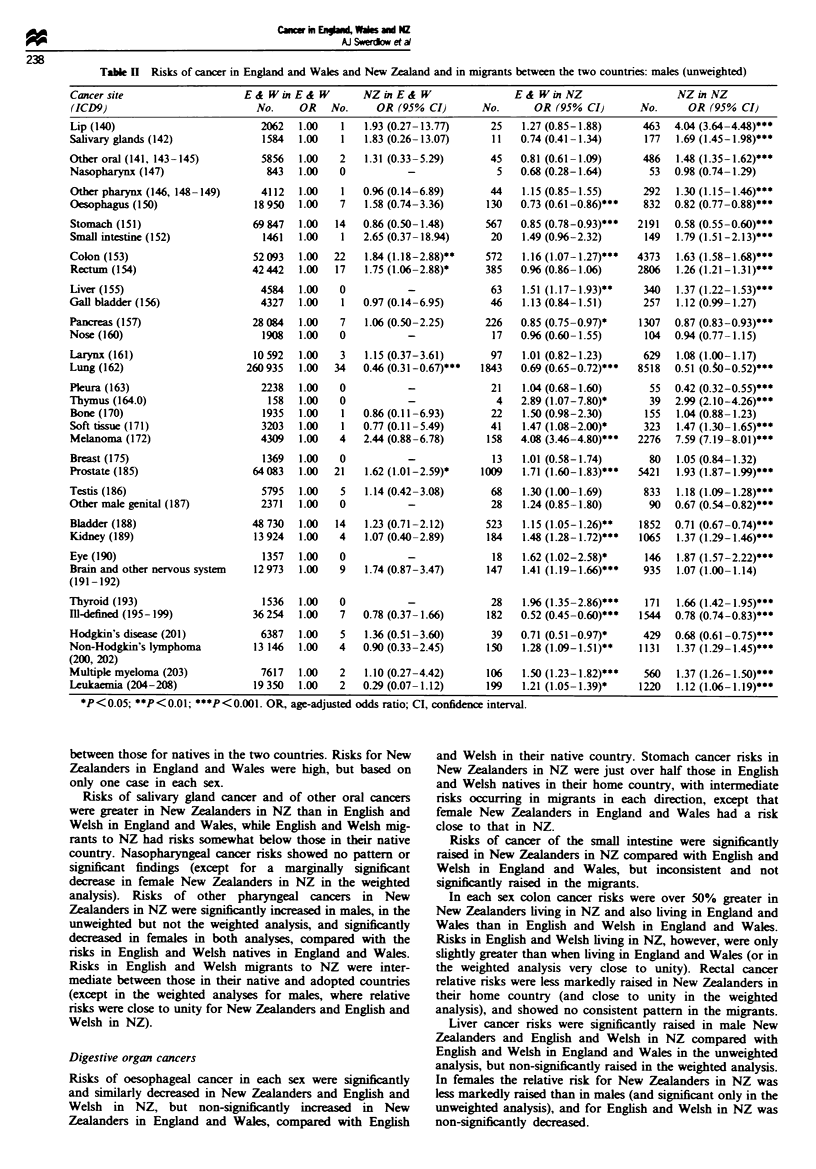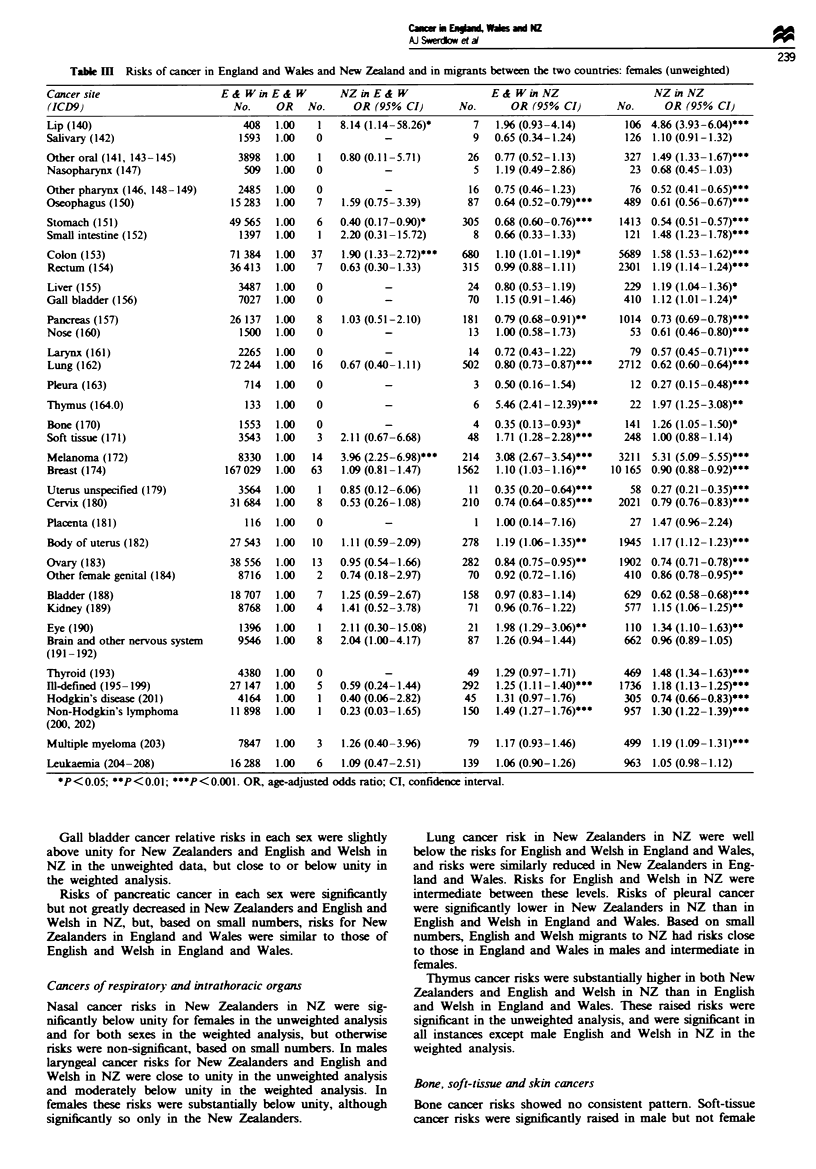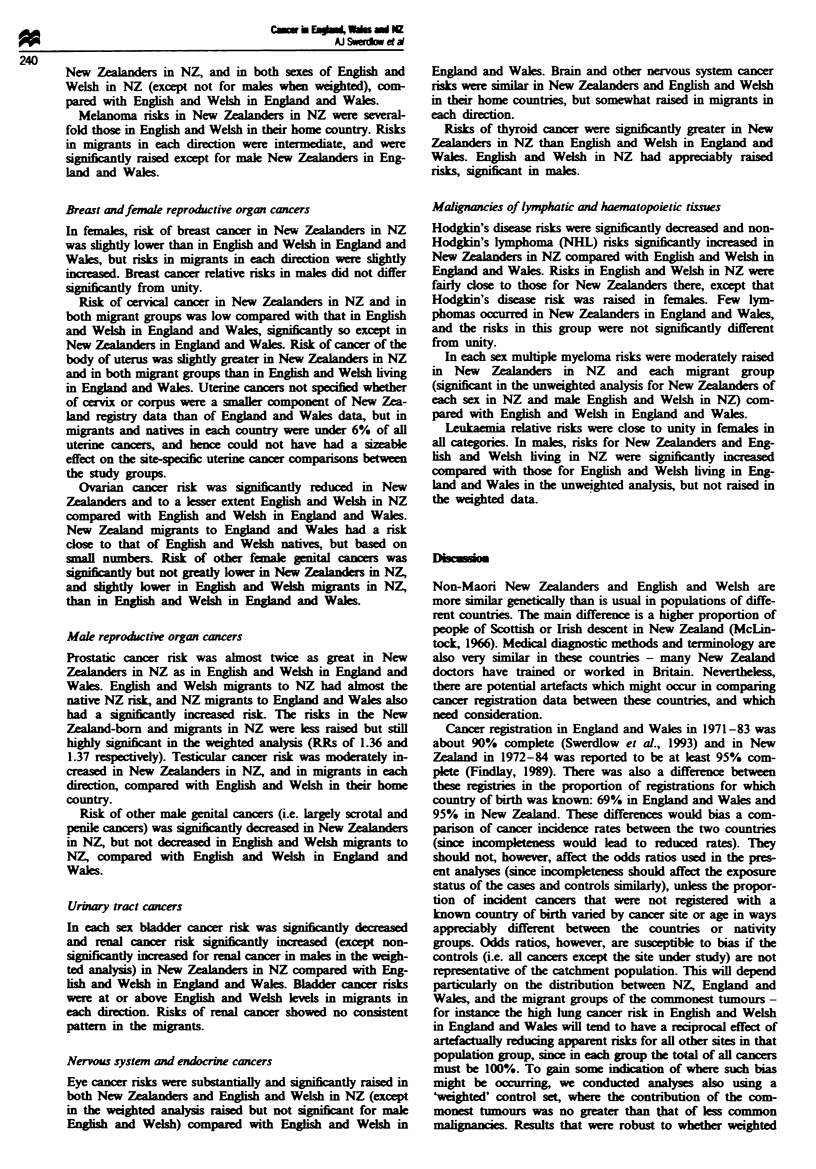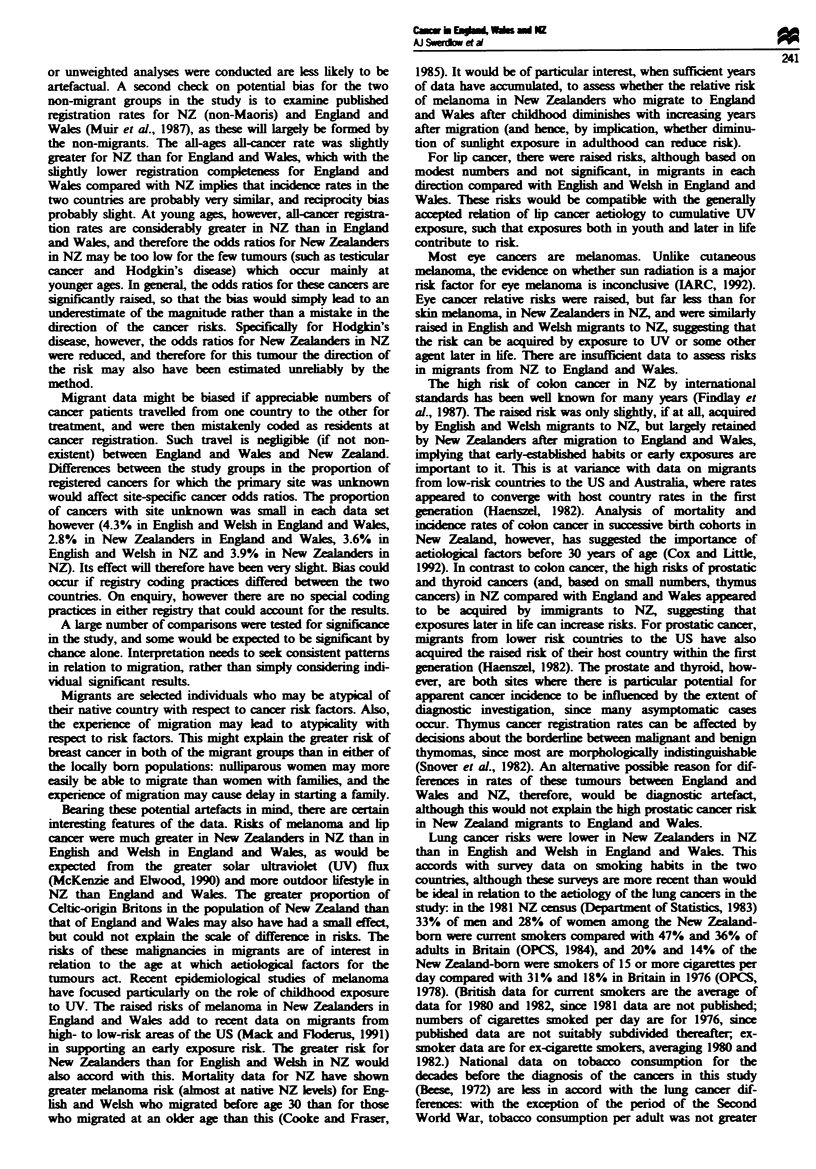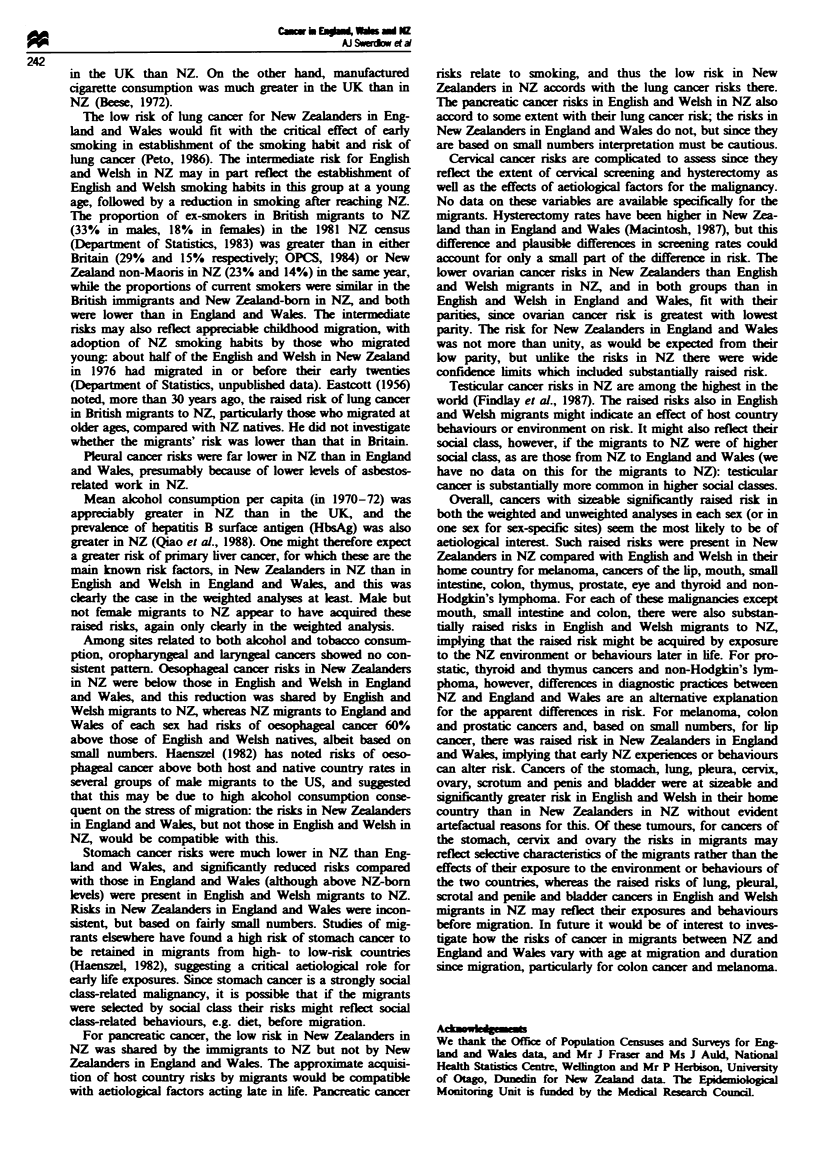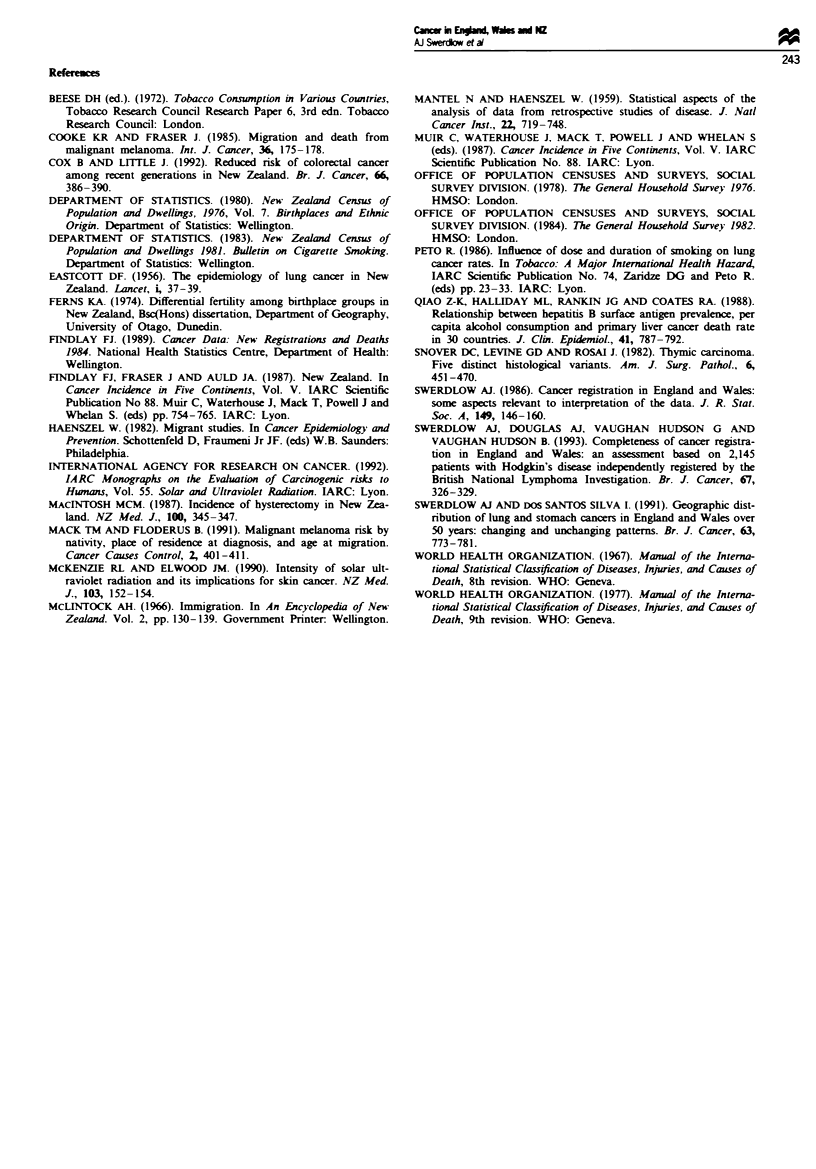# Cancer incidence in England and Wales and New Zealand and in migrants between the two countries.

**DOI:** 10.1038/bjc.1995.309

**Published:** 1995-07

**Authors:** A. J. Swerdlow, K. R. Cooke, D. C. Skegg, J. Wilkinson

**Affiliations:** Epidemiological Monitoring Unit, London School of Hygiene and Tropical Medicine, UK.

## Abstract

Risks of cancer incidence in people born in England and Wales and New Zealand (non-Maoris) living in their home countries, and after migration between the two countries, were analysed using data from their national cancer registries. Since these populations are of similar genetic origin, any real differences in cancer incidence between them are likely to reflect the action of environmental or behavioural risk factors. The greatest differences in risk between the countries were for cutaneous melanoma and lip cancer. In each sex, relative risks of these malignancies were 4 or greater for the New Zealand-born in New Zealand compared with English and Welsh natives in their home country, and risks for migrants in each direction were generally intermediate between those born in the home country in the two countries. Sizeable significantly raised risks in the New Zealand-born in New Zealand compared with English and Welsh natives in England and Wales also occurred for cancers of the mouth, small intestine, colon, thymus, eye and thyroid, and non-Hodgkin's lymphoma in each sex, and for cancer of the prostate. For all of these sites except mouth, small intestine and colon there were also risks around or above New Zealand-born levels for English and Welsh migrants to New Zealand; for colon cancer these migrants had risks close to those in England and Wales. New Zealand migrants to England and Wales had risks of cancers of the colon and prostate that were similar to or above New Zealand levels. Risks of cancers of the stomach, lung, pleura and bladder, and Hodgkin's disease in each sex, and cancers of the cervix, ovary and scrotum and penis, were substantially and significantly lower in the New Zealand-born living in New Zealand than in English and Welsh natives in England and Wales. In English and Welsh migrants to New Zealand risks of bladder cancer in each sex, and of scrotal and penile and pleural cancer in males, approximated to England and Wales risks; cervical cancer risk approximated to the New Zealand risk; and stomach, lung and ovarian cancers showed intermediate risks. Migrants from New Zealand to England and Wales did not gain the lung cancer or clearly the stomach cancer risk of their host country, but did have bladder cancer risks approximating to those in England and Wales.(ABSTRACT TRUNCATED AT 400 WORDS)


© 1995 Stockton Press All rights reserved 0007–0920/95 $12.00

### Cancer incidence in England and Wales and New Zealand and in migrants between the two countries

AJ Swerdlow[1], KR Cooke[2], DCG Skegg[2] and J Wilkinson[1]

[1]*Epidemiological Monitoring Unit, London School of Hygiene and Tropical Medicine, London, UK;* [2]*Department of Preventive and Social Medicine, University of Otago Medical School, Dunedin, New Zealand.*

**Summary** Risks of cancer incidence in people born in England and Wales and New Zealand (non-Maoris) living in their home countries, and after migration between the two countries, were analysed using data from their national cancer registries. Since these populations are of similar genetic origin, any real differences in cancer incidence between them are likely to reflect the action of environmental or behavioural risk factors. The greatest differences in risk between the countries were for cutaneous melanoma and lip cancer. In each sex, relative risks of these malignancies were 4 or greater for the New Zealand-born in New Zealand compared with English and Welsh natives in their home country, and risks for migrants in each direction were generally intermediate between those born in the home country in the two countries. Sizeable significantly raised risks in the New Zealand-born in New Zealand compared with English and Welsh natives in England and Wales also occurred for cancers of the mouth, small intestine, colon, thymus, eye and thyroid, and non-Hodgkin's lymphoma in each sex, and for cancer of the prostate. For all of these sites except mouth, small intestine and colon there were also risks around or above New Zealand-born levels for English and Welsh migrants to New Zealand; for colon cancer these migrants had risks close to those in England and Wales. New Zealand migrants to England and Wales had risks of cancers of the colon and prostate that were similar to or above New Zealand levels. Risks of cancers of the stomach, lung, pleura and bladder, and Hodgkin's disease in each sex, and cancers of the cervix, ovary and scrotum and penis, were substantially and significantly lower in the New Zealand-born living in New Zealand than in English and Welsh natives in England and Wales. In English and Welsh migrants to New Zealand risks of bladder cancer in each sex, and of scrotal and penile and pleural cancer in males, approximated to England and Wales risks; cervical cancer risk approximated to the New Zealand risk; and stomach, lung and ovarian cancers showed intermediate risks. Migrants from New Zealand to England and Wales did not gain the lung cancer or clearly the stomach cancer risk of their host country, but did have bladder cancer risks approximating to those in England and Wales. The results suggest that exposure to the New Zealand environment or behaviours early or late in life can lead to raised risks of melanoma, lip cancer and prostatic cancer; that migration to New Zealand may lead to some extent to the acquisition of raised risk of cancer of the eye and possibly of cancers of the thymus, thyroid and non-Hodgkin's lymphoma; and that exposures or behaviours early in life are critical to the high risks of colon cancer in persons born in New Zealand. Exposures early in life in England and Wales appear able to lead to raised risk of lung cancer and probably also of cancers of the pleura and scrotum and penis, and exposures early or late in life in England and Wales may raise the risk of bladder cancer.

**Keywords**: cancer incidence; migrants

Migrant studies have played an important part in demonstrating the environmental origins of many cancers, by showing sizeable changes in risk with residence in the new country (Haenszel, 1982). Migrant groups, however, are often very different genetically from the native population of the country to which they migrate, and therefore it is difficult to know whether residual differences in cancer risk between the immigrants and the natives of the host country are genetic or are due to differences in behaviour or early environment. Risks in migrants can give information on the likely age at which carcinogens act, since the early exposures of the migrants will be in their native country and the later exposures in the host country. Comparative data on migrants in both directions between two countries ought to be particularly informative, since factors acting early in life should increase risk in migrants from the high- to low-risk country but not in migrants in the opposite direction, while factors acting late should have the reverse effect. To our knowledge, however, there have been no such studies in two-way migration.

The first Europeans known to have reached New Zealand were Tasman, a Dutchman, and his crew in 1642, but the great majority of early colonists were British. Subsequent migration from Britain to New Zealand has been of such an extent that, unusually, the New Zealand non-aboriginal (non-Maori) population is largely of British descent (McLintock, 1966). There has also been appreciable migration in the opposite direction. The present study uses data from the national cancer registries of England and Wales and New Zealand to assess comparative cancer risks in residents born in the two countries and migrants between them.

Correspondence: AJ Swerdlow, Epidemiological Monitoring Unit, London School of Hygiene and Tropical Medicine, Keppel Street, London WC1E 7HT, UK
Received 15 August 1994; revised 27 January 1995; accepted 14 February 1995.

### Materials and methods

Cancer registration in England and Wales has been conducted on a national basis since 1945, with complete geographic coverage of the country since 1962 (Swerdlow, 1986). Country of birth has been recorded in the national cancer registration files since 1971. The data presented here relate to the years of incidence for which registrations were reasonably complete at the time that data were extracted for this study: 1971–83.

In New Zealand, cancer registration has been conducted nationally since 1948, and became population based in 1972 (Findlay *et al*., 1987). The data analysed in this study are for 1972–84. During this period people were generally defined as Maori if they reported half or more Maori ancestry, and the term non-Maori covered all other persons. While the non-Maori population will have included people with some Maori ancestry, the overall effect of this would not have been great because the Maori population accounted for less than 10% of the total population.

Country of birth was recorded for 95% of registrations in New Zealand, but in the England and Wales data this variable was known for only 69% of cases. Calculation of incidence rates could therefore be seriously biased, and we analysed the data instead by means of age-adjusted odds ratios (Mantel and Haenszel, 1959). These should be unaffected by incompleteness provided that it is of about the same degree for different cancer sites. The odds ratios compared the risk of each cancer in each migrant group, and in New Zealand-born people (whom we will refer to as New Zealanders) in NZ, with the risk in the England and Wales-born (whom we will refer to as English and Welsh) in England and Wales, the largest group in the study, as the baseline. The cases in these analyses were the cancer of interest (i.e. successively each cancer site) and the controls were based on all cancers except the case malignancy and non-melanoma skin cancer. The latter was excluded entirely from the analyses because it was not registered in New Zealand.

Two different methods were used to obtain the controls from among the non-case cancers. The results using these methods were then compared to help to assess whether control selection might have biased the results. Firstly, analyses were conducted using all registrations except those with the case malignancy as the controls; we have referred to these as the unweighted analyses. Secondly, analyses were conducted in which a weighted sample of all non-case registrations was used as the controls. The weighting was such that in each 5 year age group, no three-digit ICD cancer site constituted more than 5% of the controls. This was done to avoid the domination of the controls by a few common sites, such as lung cancer, whose risk would otherwise tend to affect reciprocally the apparent risk of all other tumours, since in each population the total of all cancers must be 100%. Details of the method of weighting can be found in Swerdlow and dos Santos Silva (1991).

Site of cancer was coded in the England and Wales files to ICD8 (WHO, 1967) for cancers incident in 1971–78 and to ICD9 (WHO, 1977) for 1979–83. Bridge coding was conducted, using fourth digit ICD8 data as required, to give the ICD9 categories shown in Tables II and III for the entire period. The New Zealand data were coded to ICD8 for 1972–79 and ICD9 for 1980–84, and again bridge coding was conducted to give the same ICD9 categories throughout.

The coding of New Zealanders and English and Welsh in the New Zealand data was straightforward, as was the coding of New Zealanders in the England and Wales data. Identification of English and Welsh in England and Wales was less simple, however, because of the several ways, varying in specificity, in which people born in the British Isles can have their birth place recorded in the cancer files. English and Welsh natives were taken to be those whose birth place was stated as England and Wales, or as Britain or United Kingdom not specified. The last two categories were included because they constitute about one-third of all registrations in England and Wales and are likely to be largely England and Wales-born.

To gain demographic description of the New Zealand migrants to England and Wales, for interpretation of the cancer risks, we used unpublished data and specially run tabulations from the 1971 census. At this census more detailed questions than usual were asked about migrants and about female reproductive history. We also obtained comparable New Zealand data, as far as possible, from the 1976 census, using published data (Department of Statistics, 1980) and an unpublished table previously described (Cooke and Fraser, 1985).

### Results

The native-born population of England and Wales in 1971 was 44 563 700, and the New Zealand-born non-Maori population of New Zealand in 1976 was 2 339 514. The England and Wales-born population of New Zealand in 1976 was 228 175, and the New Zealand-born population of England and Wales in 1971 was 19 680. Migration of New Zealanders to England and Wales has occurred fairly steadily over many years, being a mixture of long-term migrants and short-term students and other temporary immigrants: at the 1971 census, 20% of New Zealanders in England and Wales had arrived before 1940, 19% during 1940–59, 38% during 1960–69 and 24% during 1970–71. For the great majority of the immigrants, both parents were born in Britain (27.5%), or both parents were born in the 'Old Commonwealth' (44.0%) (the census coding category for NZ, Australia and Canada, and therefore likely to be largely New Zealanders in this instance), or one parent was born in each (21.3%). New Zealand immigrants were on average of higher social class (based on own occupation, and excluding persons of unclassified or unknown class) than English and Welsh natives in England and Wales: 59% of New Zealand-born men and 41% of New Zealand-born wormen were in classes I and II compared with 24% of men and 17% of women among English and Welsh natives. Thirty-three per cent of New Zealand female immigrants aged 40–59 were single (15.9%) or married but nulliparous (17.2%), and the mean parity of these migrants was 1.63, compared with 20.7% who were either single (8.1%) or married nulliparous (12.6%), and a mean parity of 1.89, among England and Wales native women of the same age. Parity was only recorded at the census for children born within marriage. We have therefore had to assume for the calculation of parity that all single women were nulliparous.

Migration from England and Wales to New Zealand has occurred over many decades, but the number of migrants increased after the Second World War: at the 1976 New Zealand census, 18% of English and Welsh in New Zealand had arrived before 1947, 29% during 1947–61, 26% during 1962–71 and 28% during 1972–76, when there was a short-lived but large increase in the inflow of British immigrants. Immigrants from England and Wales had higher fertility rates (live births per 1000 women aged 15–44 years) than women in England and Wales and lower rates than New Zealand-born non-Maori women. Much of the difference between New Zealand-born non-Maori women and English and Welsh-born women in New Zealand, however, is attributable to age differences, as age-standardised fertility ratios were similar for the two groups (Ferns, 1974). Data are not available on the social class composition of migrants to New Zealand during the relevant period.

The numbers of cancers on which the analyses of cancer risk were based are shown in Table I. There were large numbers for the native populations of New Zealand and England and Wales in their home countries, and for the England and Wales-born in New Zealand, but far fewer for New Zealanders in England and Wales, with consequent lower precision of the cancer risk estimates for this group.

Tables II and III show risks of cancer in the migrant groups, and in New Zealanders in New Zealand, compared with English and Welsh in England and Wales. The data presented are from the 'unweighted' analyses (see Materials and methods). Where the 'weighted' analyses gave substantially different results from these, they are quoted in the text.

### *Lip, oral and pharyngeal cancers*

Risks of lip cancer were 4- to 5-fold significantly increased in each sex in New Zealanders in New Zealand, compared with English and Welsh in England and Wales. Risks for English and Welsh migrants to New Zealand were intermediate

**Table I** Numbers of cancers incident in English and Welsh and New Zealanders in England and Wales 1971–83 and New Zealand 1972–84, by country of birth and country of residence

| | *Number of cancers incident* | |
|---|---|---|
| | *Males* | *Females* |
| New Zealanders in NZ | 41 488 | 42 390 |
| English and Welsh in NZ | 7060 | 6072 |
| New Zealanders in England and Wales | 193 | 236 |
| English and Welsh in England and Wales | 759 495 | 701 302 |

Table II Risks of cancer in England and Wales and New Zealand and in migrants between the two countries: males (unweighted)

| Cancer site (ICD9) | E & W in E & W No. | E & W in E & W OR | NZ in E & W No. | NZ in E & W OR (95% CI) | E & W in NZ No. | E & W in NZ OR (95% CI) | NZ in NZ No. | NZ in NZ OR (95% CI) |
|---|---|---|---|---|---|---|---|---|
| Lip (140) | 2062 | 1.00 | 1 | 1.93 (0.27-13.77) | 25 | 1.27 (0.85-1.88) | 463 | 4.04 (3.64-4.48)*** |
| Salivary glands (142) | 1584 | 1.00 | 1 | 1.83 (0.26-13.07) | 11 | 0.74 (0.41-1.34) | 177 | 1.69 (1.45-1.98)*** |
| Other oral (141, 143-145) | 5856 | 1.00 | 2 | 1.31 (0.33-5.29) | 45 | 0.81 (0.61-1.09) | 486 | 1.48 (1.35-1.62)*** |
| Nasopharynx (147) | 843 | 1.00 | 0 | – | 5 | 0.68 (0.28-1.64) | 53 | 0.98 (0.74-1.29) |
| Other pharynx (146, 148-149) | 4112 | 1.00 | 1 | 0.96 (0.14-6.89) | 44 | 1.15 (0.85-1.55) | 292 | 1.30 (1.15-1.46)*** |
| Oesophagus (150) | 18 950 | 1.00 | 7 | 1.58 (0.74-3.36) | 130 | 0.73 (0.61-0.86)*** | 832 | 0.82 (0.77-0.88)*** |
| Stomach (151) | 69 847 | 1.00 | 14 | 0.86 (0.50-1.48) | 567 | 0.85 (0.78-0.93)*** | 2191 | 0.58 (0.55-0.60)*** |
| Small intestine (152) | 1461 | 1.00 | 1 | 2.65 (0.37-18.94) | 20 | 1.49 (0.96-2.32) | 149 | 1.79 (1.51-2.13)*** |
| Colon (153) | 52 093 | 1.00 | 22 | 1.84 (1.18-2.88)** | 572 | 1.16 (1.07-1.27)*** | 4373 | 1.63 (1.58-1.68)*** |
| Rectum (154) | 42 442 | 1.00 | 17 | 1.75 (1.06-2.88)* | 385 | 0.96 (0.86-1.06) | 2806 | 1.26 (1.21-1.31)*** |
| Liver (155) | 4584 | 1.00 | 0 | – | 63 | 1.51 (1.17-1.93)** | 340 | 1.37 (1.22-1.53)*** |
| Gall bladder (156) | 4327 | 1.00 | 1 | 0.97 (0.14-6.95) | 46 | 1.13 (0.84-1.51) | 257 | 1.12 (0.99-1.27) |
| Pancreas (157) | 28 084 | 1.00 | 7 | 1.06 (0.50-2.25) | 226 | 0.85 (0.75-0.97)* | 1307 | 0.87 (0.83-0.93)*** |
| Nose (160) | 1908 | 1.00 | 0 | – | 17 | 0.96 (0.60-1.55) | 104 | 0.94 (0.77-1.15) |
| Larynx (161) | 10 592 | 1.00 | 3 | 1.15 (0.37-3.61) | 97 | 1.01 (0.82-1.23) | 629 | 1.08 (1.00-1.17) |
| Lung (162) | 260 935 | 1.00 | 34 | 0.46 (0.31-0.67)*** | 1843 | 0.69 (0.65-0.72)*** | 8518 | 0.51 (0.50-0.52)*** |
| Pleura (163) | 2238 | 1.00 | 0 | – | 21 | 1.04 (0.68-1.60) | 55 | 0.42 (0.32-0.55)*** |
| Thymus (164.0) | 158 | 1.00 | 0 | – | 4 | 2.89 (1.07-7.80)* | 39 | 2.99 (2.10-4.26)*** |
| Bone (170) | 1935 | 1.00 | 1 | 0.86 (0.11-6.93) | 22 | 1.50 (0.98-2.30) | 155 | 1.04 (0.88-1.23) |
| Soft tissue (171) | 3203 | 1.00 | 1 | 0.77 (0.11-5.49) | 41 | 1.47 (1.08-2.00)* | 323 | 1.47 (1.30-1.65)*** |
| Melanoma (172) | 4309 | 1.00 | 4 | 2.44 (0.88-6.78) | 158 | 4.08 (3.46-4.80)*** | 2276 | 7.59 (7.19-8.01)*** |
| Breast (175) | 1369 | 1.00 | 0 | – | 13 | 1.01 (0.58-1.74) | 80 | 1.05 (0.84-1.32) |
| Prostate (185) | 64 083 | 1.00 | 21 | 1.62 (1.01-2.59)* | 1009 | 1.71 (1.60-1.83)*** | 5421 | 1.93 (1.87-1.99)*** |
| Testis (186) | 5795 | 1.00 | 5 | 1.14 (0.42-3.08) | 68 | 1.30 (1.00-1.69) | 833 | 1.18 (1.09-1.28)*** |
| Other male genital (187) | 2371 | 1.00 | 0 | – | 28 | 1.24 (0.85-1.80) | 90 | 0.67 (0.54-0.82)*** |
| Bladder (188) | 48 730 | 1.00 | 14 | 1.23 (0.71-2.12) | 523 | 1.15 (1.05-1.26)** | 1852 | 0.71 (0.67-0.74)*** |
| Kidney (189) | 13 924 | 1.00 | 4 | 1.07 (0.40-2.89) | 184 | 1.48 (1.28-1.72)*** | 1065 | 1.37 (1.29-1.46)*** |
| Eye (190) | 1357 | 1.00 | 0 | – | 18 | 1.62 (1.02-2.58)* | 146 | 1.87 (1.57-2.22)*** |
| Brain and other nervous system (191-192) | 12 973 | 1.00 | 9 | 1.74 (0.87-3.47) | 147 | 1.41 (1.19-1.66)*** | 935 | 1.07 (1.00-1.14) |
| Thyroid (193) | 1536 | 1.00 | 0 | – | 28 | 1.96 (1.35-2.86)*** | 171 | 1.66 (1.42-1.95)*** |
| Ill-defined (195-199) | 36 254 | 1.00 | 7 | 0.78 (0.37-1.66) | 182 | 0.52 (0.45-0.60)*** | 1544 | 0.78 (0.74-0.83)*** |
| Hodgkin's disease (201) | 6387 | 1.00 | 5 | 1.36 (0.51-3.60) | 39 | 0.71 (0.51-0.97)* | 429 | 0.68 (0.61-0.75)*** |
| Non-Hodgkin's lymphoma (200, 202) | 13 146 | 1.00 | 4 | 0.90 (0.33-2.45) | 150 | 1.28 (1.09-1.51)** | 1131 | 1.37 (1.29-1.45)*** |
| Multiple myeloma (203) | 7617 | 1.00 | 2 | 1.10 (0.27-4.42) | 106 | 1.50 (1.23-1.82)*** | 560 | 1.37 (1.26-1.50)*** |
| Leukaemia (204-208) | 19 350 | 1.00 | 2 | 0.29 (0.07-1.12) | 199 | 1.21 (1.05-1.39)* | 1220 | 1.12 (1.06-1.19)*** |

*$P<0.05$; **$P<0.01$; ***$P<0.001$. OR, age-adjusted odds ratio; CI, confidence interval.

between those for natives in the two countries. Risks for New Zealanders in England and Wales were high, but based on only one case in each sex.

Risks of salivary gland cancer and of other oral cancers were greater in New Zealanders in NZ than in English and Welsh in England and Wales, while English and Welsh migrants to NZ had risks somewhat below those in their native country. Nasopharyngeal cancer risks showed no pattern or significant findings (except for a marginally significant decrease in female New Zealanders in NZ in the weighted analysis). Risks of other pharyngeal cancers in New Zealanders in NZ were significantly increased in males, in the unweighted but not the weighted analysis, and significantly decreased in females in both analyses, compared with the risks in English and Welsh natives in England and Wales. Risks in English and Welsh migrants to NZ were intermediate between those in their native and adopted countries (except in the weighted analyses for males, where relative risks were close to unity for New Zealanders and English and Welsh in NZ).

### *Digestive organ cancers*

Risks of oesophageal cancer in each sex were significantly and similarly decreased in New Zealanders and English and Welsh in NZ, but non-significantly increased in New Zealanders in England and Wales, compared with English and Welsh in their native country. Stomach cancer risks in New Zealanders in NZ were just over half those in English and Welsh natives in their home country, with intermediate risks occurring in migrants in each direction, except that female New Zealanders in England and Wales had a risk close to that in NZ.

Risks of cancer of the small intestine were significantly raised in New Zealanders in NZ compared with English and Welsh in England and Wales, but inconsistent and not significantly raised in the migrants.

In each sex colon cancer risks were over 50% greater in New Zealanders living in NZ and also living in England and Wales than in English and Welsh in England and Wales. Risks in English and Welsh living in NZ, however, were only slightly greater than when living in England and Wales (or in the weighted analysis very close to unity). Rectal cancer relative risks were less markedly raised in New Zealanders in their home country (and close to unity in the weighted analysis), and showed no consistent pattern in the migrants.

Liver cancer risks were significantly raised in male New Zealanders and English and Welsh in NZ compared with English and Welsh in England and Wales in the unweighted analysis, but non-significantly raised in the weighted analysis. In females the relative risk for New Zealanders in NZ was less markedly raised than in males (and significant only in the unweighted analysis), and for English and Welsh in NZ was non-significantly decreased.

Table III Risks of cancer in England and Wales and New Zealand and in migrants between the two countries: females (unweighted)

| Cancer site (ICD9) | E & W in E & W No. | E & W in E & W OR | NZ in E & W No. | NZ in E & W OR (95% CI) | E & W in NZ No. | E & W in NZ OR (95% CI) | NZ in NZ No. | NZ in NZ OR (95% CI) |
|---|---|---|---|---|---|---|---|---|
| Lip (140) | 408 | 1.00 | 1 | 8.14 (1.14-58.26)* | 7 | 1.96 (0.93-4.14) | 106 | 4.86 (3.93-6.04)*** |
| Salivary (142) | 1593 | 1.00 | 0 | – | 9 | 0.65 (0.34-1.24) | 126 | 1.10 (0.91-1.32) |
| Other oral (141, 143-145) | 3898 | 1.00 | 1 | 0.80 (0.11-5.71) | 26 | 0.77 (0.52-1.13) | 327 | 1.49 (1.33-1.67)*** |
| Nasopharynx (147) | 509 | 1.00 | 0 | – | 5 | 1.19 (0.49-2.86) | 23 | 0.68 (0.45-1.03) |
| Other pharynx (146, 148-149) | 2485 | 1.00 | 0 | – | 16 | 0.75 (0.46-1.23) | 76 | 0.52 (0.41-0.65)*** |
| Oseophagus (150) | 15 283 | 1.00 | 7 | 1.59 (0.75-3.39) | 87 | 0.64 (0.52-0.79)*** | 489 | 0.61 (0.56-0.67)*** |
| Stomach (151) | 49 565 | 1.00 | 6 | 0.40 (0.17-0.90)* | 305 | 0.68 (0.60-0.76)*** | 1413 | 0.54 (0.51-0.57)*** |
| Small intestine (152) | 1397 | 1.00 | 1 | 2.20 (0.31-15.72) | 8 | 0.66 (0.33-1.33) | 121 | 1.48 (1.23-1.78)*** |
| Colon (153) | 71 384 | 1.00 | 37 | 1.90 (1.33-2.72)*** | 680 | 1.10 (1.01-1.19)* | 5689 | 1.58 (1.53-1.62)*** |
| Rectum (154) | 36 413 | 1.00 | 7 | 0.63 (0.30-1.33) | 315 | 0.99 (0.88-1.11) | 2301 | 1.19 (1.14-1.24)*** |
| Liver (155) | 3487 | 1.00 | 0 | – | 24 | 0.80 (0.53-1.19) | 229 | 1.19 (1.04-1.36)* |
| Gall bladder (156) | 7027 | 1.00 | 0 | – | 70 | 1.15 (0.91-1.46) | 410 | 1.12 (1.01-1.24)* |
| Pancreas (157) | 26 137 | 1.00 | 8 | 1.03 (0.51-2.10) | 181 | 0.79 (0.68-0.91)** | 1014 | 0.73 (0.69-0.78)*** |
| Nose (160) | 1500 | 1.00 | 0 | – | 13 | 1.00 (0.58-1.73) | 53 | 0.61 (0.46-0.80)*** |
| Larynx (161) | 2265 | 1.00 | 0 | – | 14 | 0.72 (0.43-1.22) | 79 | 0.57 (0.45-0.71)*** |
| Lung (162) | 72 244 | 1.00 | 16 | 0.67 (0.40-1.11) | 502 | 0.80 (0.73-0.87)*** | 2712 | 0.62 (0.60-0.64)*** |
| Pleura (163) | 714 | 1.00 | 0 | – | 3 | 0.50 (0.16-1.54) | 12 | 0.27 (0.15-0.48)*** |
| Thymus (164.0) | 133 | 1.00 | 0 | – | 6 | 5.46 (2.41-12.39)*** | 22 | 1.97 (1.25-3.08)** |
| Bone (170) | 1553 | 1.00 | 0 | – | 4 | 0.35 (0.13-0.93)* | 141 | 1.26 (1.05-1.50)* |
| Soft tissue (171) | 3543 | 1.00 | 3 | 2.11 (0.67-6.68) | 48 | 1.71 (1.28-2.28)*** | 248 | 1.00 (0.88-1.14) |
| Melanoma (172) | 8330 | 1.00 | 14 | 3.96 (2.25-6.98)*** | 214 | 3.08 (2.67-3.54)*** | 3211 | 5.31 (5.09-5.55)*** |
| Breast (174) | 167 029 | 1.00 | 63 | 1.09 (0.81-1.47) | 1562 | 1.10 (1.03-1.16)** | 10 165 | 0.90 (0.88-0.92)*** |
| Uterus unspecified (179) | 3564 | 1.00 | 1 | 0.85 (0.12-6.06) | 11 | 0.35 (0.20-0.64)*** | 58 | 0.27 (0.21-0.35)*** |
| Cervix (180) | 31 684 | 1.00 | 8 | 0.53 (0.26-1.08) | 210 | 0.74 (0.64-0.85)*** | 2021 | 0.79 (0.76-0.83)*** |
| Placenta (181) | 116 | 1.00 | 0 | – | 1 | 1.00 (0.14-7.16) | 27 | 1.47 (0.96-2.24) |
| Body of uterus (182) | 27 543 | 1.00 | 10 | 1.11 (0.59-2.09) | 278 | 1.19 (1.06-1.35)** | 1945 | 1.17 (1.12-1.23)*** |
| Ovary (183) | 38 556 | 1.00 | 13 | 0.95 (0.54-1.66) | 282 | 0.84 (0.75-0.95)** | 1902 | 0.74 (0.71-0.78)*** |
| Other female genital (184) | 8716 | 1.00 | 2 | 0.74 (0.18-2.97) | 70 | 0.92 (0.72-1.16) | 410 | 0.86 (0.78-0.95)** |
| Bladder (188) | 18 707 | 1.00 | 7 | 1.25 (0.59-2.67) | 158 | 0.97 (0.83-1.14) | 629 | 0.62 (0.58-0.68)*** |
| Kidney (189) | 8768 | 1.00 | 4 | 1.41 (0.52-3.78) | 71 | 0.96 (0.76-1.22) | 577 | 1.15 (1.06-1.25)** |
| Eye (190) | 1396 | 1.00 | 1 | 2.11 (0.30-15.08) | 21 | 1.98 (1.29-3.06)** | 110 | 1.34 (1.10-1.63)** |
| Brain and other nervous system (191-192) | 9546 | 1.00 | 8 | 2.04 (1.00-4.17) | 87 | 1.26 (0.94-1.44) | 662 | 0.96 (0.89-1.05) |
| Thyroid (193) | 4380 | 1.00 | 0 | – | 49 | 1.29 (0.97-1.71) | 469 | 1.48 (1.34-1.63)*** |
| Ill-defined (195-199) | 27 147 | 1.00 | 5 | 0.59 (0.24-1.44) | 292 | 1.25 (1.11-1.40)*** | 1736 | 1.18 (1.13-1.25)*** |
| Hodgkin's disease (201) | 4164 | 1.00 | 1 | 0.40 (0.06-2.82) | 45 | 1.31 (0.97-1.76) | 305 | 0.74 (0.66-0.83)*** |
| Non-Hodgkin's lymphoma (200, 202) | 11 898 | 1.00 | 1 | 0.23 (0.03-1.65) | 150 | 1.49 (1.27-1.76)*** | 957 | 1.30 (1.22-1.39)*** |
| Multiple myeloma (203) | 7847 | 1.00 | 3 | 1.26 (0.40-3.96) | 79 | 1.17 (0.93-1.46) | 499 | 1.19 (1.09-1.31)*** |
| Leukaemia (204-208) | 16 288 | 1.00 | 6 | 1.09 (0.47-2.51) | 139 | 1.06 (0.90-1.26) | 963 | 1.05 (0.98-1.12) |

$^{*}P<0.05$; $^{**}P<0.01$; $^{***}P<0.001$. OR, age-adjusted odds ratio; CI, confidence interval.

Gall bladder cancer relative risks in each sex were slightly above unity for New Zealanders and English and Welsh in NZ in the unweighted data, but close to or below unity in the weighted analysis.

Risks of pancreatic cancer in each sex were significantly but not greatly decreased in New Zealanders and English and Welsh in NZ, but, based on small numbers, risks for New Zealanders in England and Wales were similar to those of English and Welsh in England and Wales.

### *Cancers of respiratory and intrathoracic organs*

Nasal cancer risks in New Zealanders in NZ were significantly below unity for females in the unweighted analysis and for both sexes in the weighted analysis, but otherwise risks were non-significant, based on small numbers. In males laryngeal cancer risks for New Zealanders and English and Welsh in NZ were close to unity in the unweighted analysis and moderately below unity in the weighted analysis. In females these risks were substantially below unity, although significantly so only in the New Zealanders.

Lung cancer risk in New Zealanders in NZ were well below the risks for English and Welsh in England and Wales, and risks were similarly reduced in New Zealanders in England and Wales. Risks for English and Welsh in NZ were intermediate between these levels. Risks of pleural cancer were significantly lower in New Zealanders in NZ than in English and Welsh in England and Wales. Based on small numbers, English and Welsh migrants to NZ had risks close to those in England and Wales in males and intermediate in females.

Thymus cancer risks were substantially higher in both New Zealanders and English and Welsh in NZ than in English and Welsh in England and Wales. These raised risks were significant in the unweighted analysis, and were significant in all instances except male English and Welsh in NZ in the weighted analysis.

### *Bone, soft-tissue and skin cancers*

Bone cancer risks showed no consistent pattern. Soft-tissue cancer risks were significantly raised in male but not female

New Zealanders in NZ, and in both sexes of English and Welsh in NZ (except not for males when weighted), compared with English and Welsh in England and Wales.

Melanoma risks in New Zealanders in NZ were severalfold those in English and Welsh in their home country. Risks in migrants in each direction were intermediate, and were significantly raised except for male New Zealanders in England and Wales.

*Breast and female reproductive organ cancers*

In females, risk of breast cancer in New Zealanders in NZ was slightly lower than in English and Welsh in England and Wales, but risks in migrants in each direction were slightly increased. Breast cancer relative risks in males did not differ significantly from unity.

Risk of cervical cancer in New Zealanders in NZ and in both migrant groups was low compared with that in English and Welsh in England and Wales, significantly so except in New Zealanders in England and Wales. Risk of cancer of the body of uterus was slightly greater in New Zealanders in NZ and in both migrant groups than in English and Welsh living in England and Wales. Uterine cancers not specified whether of cervix or corpus were a smaller component of New Zealand registry data than of England and Wales data, but in migrants and natives in each country were under 6% of all uterine cancers, and hence could not have had a sizeable effect on the site-specific uterine cancer comparisons between the study groups.

Ovarian cancer risk was significantly reduced in New Zealanders and to a lesser extent English and Welsh in NZ compared with English and Welsh in England and Wales. New Zealand migrants to England and Wales had a risk close to that of English and Welsh natives, but based on small numbers. Risk of other female genital cancers was significantly but not greatly lower in New Zealanders in NZ, and slightly lower in English and Welsh migrants in NZ, than in English and Welsh in England and Wales.

*Male reproductive organ cancers*

Prostatic cancer risk was almost twice as great in New Zealanders in NZ as in English and Welsh in England and Wales. English and Welsh migrants to NZ had almost the native NZ risk, and NZ migrants to England and Wales also had a significantly increased risk. The risks in the New Zealand-born and migrants in NZ were less raised but still highly significant in the weighted analysis (RRs of 1.36 and 1.37 respectively). Testicular cancer risk was moderately increased in New Zealanders in NZ, and in migrants in each direction, compared with English and Welsh in their home country.

Risk of other male genital cancers (i.e. largely scrotal and penile cancers) was significantly decreased in New Zealanders in NZ, but not decreased in English and Welsh migrants to NZ, compared with English and Welsh in England and Wales.

*Urinary tract cancers*

In each sex bladder cancer risk was significantly decreased and renal cancer risk significantly increased (except non-significantly increased for renal cancer in males in the weighted analysis) in New Zealanders in NZ compared with English and Welsh in England and Wales. Bladder cancer risks were at or above English and Welsh levels in migrants in each direction. Risks of renal cancer showed no consistent pattern in the migrants.

*Nervous system and endocrine cancers*

Eye cancer risks were substantially and significantly raised in both New Zealanders and English and Welsh in NZ (except in the weighted analysis raised but not significant for male English and Welsh) compared with English and Welsh in England and Wales. Brain and other nervous system cancer risks were similar in New Zealanders and English and Welsh in their home countries, but somewhat raised in migrants in each direction.

Risks of thyroid cancer were significantly greater in New Zealanders in NZ than English and Welsh in England and Wales. English and Welsh in NZ had appreciably raised risks, significant in males.

*Malignancies of lymphatic and haematopoietic tissues*

Hodgkin's disease risks were significantly decreased and non-Hodgkin's lymphoma (NHL) risks significantly increased in New Zealanders in NZ compared with English and Welsh in England and Wales. Risks in English and Welsh in NZ were fairly close to those for New Zealanders there, except that Hodgkin's disease risk was raised in females. Few lymphomas occurred in New Zealanders in England and Wales, and the risks in this group were not significantly different from unity.

In each sex multiple myeloma risks were moderately raised in New Zealanders in NZ and each migrant group (significant in the unweighted analysis for New Zealanders of each sex in NZ and male English and Welsh in NZ) compared with English and Welsh in England and Wales.

Leukaemia relative risks were close to unity in females in all categories. In males, risks for New Zealanders and English and Welsh living in NZ were significantly increased compared with those for English and Welsh living in England and Wales in the unweighted analysis, but not raised in the weighted data.

### Discussion

Non-Maori New Zealanders and English and Welsh are more similar genetically than is usual in populations of different countries. The main difference is a higher proportion of people of Scottish or Irish descent in New Zealand (McLintock, 1966). Medical diagnostic methods and terminology are also very similar in these countries – many New Zealand doctors have trained or worked in Britain. Nevertheless, there are potential artefacts which might occur in comparing cancer registration data between these countries, and which need consideration.

Cancer registration in England and Wales in 1971–83 was about 90% complete (Swerdlow *et al.*, 1993) and in New Zealand in 1972–84 was reported to be at least 95% complete (Findlay, 1989). There was also a difference between these registries in the proportion of registrations for which country of birth was known: 69% in England and Wales and 95% in New Zealand. These differences would bias a comparison of cancer incidence rates between the two countries (since incompleteness would lead to reduced rates). They should not, however, affect the odds ratios used in the present analyses (since incompleteness should affect the exposure status of the cases and controls similarly), unless the proportion of incident cancers that were not registered with a known country of birth varied by cancer site or age in ways appreciably different between the countries or nativity groups. Odds ratios, however, are susceptible to bias if the controls (i.e. all cancers except the site under study) are not representative of the catchment population. This will depend particularly on the distribution between NZ, England and Wales, and the migrant groups of the commonest tumours – for instance the high lung cancer risk in English and Welsh in England and Wales will tend to have a reciprocal effect of artefactually reducing apparent risks for all other sites in that population group, since in each group the total of all cancers must be 100%. To gain some indication of where such bias might be occurring, we conducted analyses also using a 'weighted' control set, where the contribution of the commonest tumours was no greater than that of less common malignancies. Results that were robust to whether weighted or unweighted analyses were conducted are less likely to be artefactual. A second check on potential bias for the two non-migrant groups in the study is to examine published registration rates for NZ (non-Maoris) and England and Wales (Muir *et al.*, 1987), as these will largely be formed by the non-migrants. The all-ages all-cancer rate was slightly greater for NZ than for England and Wales, which with the slightly lower registration completeness for England and Wales compared with NZ implies that incidence rates in the two countries are probably very similar, and reciprocity bias probably slight. At young ages, however, all-cancer registration rates are considerably greater in NZ than in England and Wales, and therefore the odds ratios for New Zealanders in NZ may be too low for the few tumours (such as testicular cancer and Hodgkin's disease) which occur mainly at younger ages. In general, the odds ratios for these cancers are significantly raised, so that the bias would simply lead to an underestimate of the magnitude rather than a mistake in the direction of the cancer risks. Specifically for Hodgkin's disease, however, the odds ratios for New Zealanders in NZ were reduced, and therefore for this tumour the direction of the risk may also have been estimated unreliably by the method.

Migrant data might be biased if appreciable numbers of cancer patients travelled from one country to the other for treatment, and were then mistakenly coded as residents at cancer registration. Such travel is negligible (if not non-existent) between England and Wales and New Zealand. Differences between the study groups in the proportion of registered cancers for which the primary site was unknown would affect site-specific cancer odds ratios. The proportion of cancers with site unknown was small in each data set however (4.3% in English and Welsh in England and Wales, 2.8% in New Zealanders in England and Wales, 3.6% in English and Welsh in NZ and 3.9% in New Zealanders in NZ). Its effect will therefore have been very slight. Bias could occur if registry coding practices differed between the two countries. On enquiry, however there are no special coding practices in either registry that could account for the results.

A large number of comparisons were tested for significance in the study, and some would be expected to be significant by chance alone. Interpretation needs to seek consistent patterns in relation to migration, rather than simply considering individual significant results.

Migrants are selected individuals who may be atypical of their native country with respect to cancer risk factors. Also, the experience of migration may lead to atypicality with respect to risk factors. This might explain the greater risk of breast cancer in both of the migrant groups than in either of the locally born populations: nulliparous women may more easily be able to migrate than women with families, and the experience of migration may cause delay in starting a family.

Bearing these potential artefacts in mind, there are certain interesting features of the data. Risks of melanoma and lip cancer were much greater in New Zealanders in NZ than in English and Welsh in England and Wales, as would be expected from the greater solar ultraviolet (UV) flux (McKenzie and Elwood, 1990) and more outdoor lifestyle in NZ than England and Wales. The greater proportion of Celtic-origin Britons in the population of New Zealand than that of England and Wales may also have had a small effect, but could not explain the scale of difference in risks. The risks of these malignancies in migrants are of interest in relation to the age at which aetiological factors for the tumours act. Recent epidemiological studies of melanoma have focused particularly on the role of childhood exposure to UV. The raised risks of melanoma in New Zealanders in England and Wales add to recent data on migrants from high- to low-risk areas of the US (Mack and Floderus, 1991) in supporting an early exposure risk. The greater risk for New Zealanders than for English and Welsh in NZ would also accord with this. Mortality data for NZ have shown greater melanoma risk (almost at native NZ levels) for English and Welsh who migrated before age 30 than for those who migrated at an older age than this (Cooke and Fraser, 1985). It would be of particular interest, when sufficient years of data have accumulated, to assess whether the relative risk of melanoma in New Zealanders who migrate to England and Wales after childhood diminishes with increasing years after migration (and hence, by implication, whether diminution of sunlight exposure in adulthood can reduce risk).

For lip cancer, there were raised risks, although based on modest numbers and not significant, in migrants in each direction compared with English and Welsh in England and Wales. These risks would be compatible with the generally accepted relation of lip cancer aetiology to cumulative UV exposure, such that exposures both in youth and later in life contribute to risk.

Most eye cancers are melanomas. Unlike cutaneous melanoma, the evidence on whether sun radiation is a major risk factor for eye melanoma is inconclusive (IARC, 1992). Eye cancer relative risks were raised, but far less than for skin melanoma, in New Zealanders in NZ, and were similarly raised in English and Welsh migrants to NZ, suggesting that the risk can be acquired by exposure to UV or some other agent later in life. There are insufficient data to assess risks in migrants from NZ to England and Wales.

The high risk of colon cancer in NZ by international standards has been well known for many years (Findlay *et al.*, 1987). The raised risk was only slightly, if at all, acquired by English and Welsh migrants to NZ, but largely retained by New Zealanders after migration to England and Wales, implying that early-established habits or early exposures are important to it. This is at variance with data on migrants from low-risk countries to the US and Australia, where rates appeared to converge with host country rates in the first generation (Haenszel, 1982). Analysis of mortality and incidence rates of colon cancer in successive birth cohorts in New Zealand, however, has suggested the importance of aetiological factors before 30 years of age (Cox and Little, 1992). In contrast to colon cancer, the high risks of prostatic and thyroid cancers (and, based on small numbers, thymus cancers) in NZ compared with England and Wales appeared to be acquired by immigrants to NZ, suggesting that exposures later in life can increase risks. For prostatic cancer, migrants from lower risk countries to the US have also acquired the raised risk of their host country within the first generation (Haenszel, 1982). The prostate and thyroid, however, are both sites where there is particular potential for apparent cancer incidence to be influenced by the extent of diagnostic investigation, since many asymptomatic cases occur. Thymus cancer registration rates can be affected by decisions about the borderline between malignant and benign thymomas, since most are morphologically indistinguishable (Snover *et al.*, 1982). An alternative possible reason for differences in rates of these tumours between England and Wales and NZ, therefore, would be diagnostic artefact, although this would not explain the high prostatic cancer risk in New Zealand migrants to England and Wales.

Lung cancer risks were lower in New Zealanders in NZ than in English and Welsh in England and Wales. This accords with survey data on smoking habits in the two countries, although these surveys are more recent than would be ideal in relation to the aetiology of the lung cancers in the study: in the 1981 NZ census (Department of Statistics, 1983) 33% of men and 28% of women among the New Zealand-born were current smokers compared with 47% and 36% of adults in Britain (OPCS, 1984), and 20% and 14% of the New Zealand-born were smokers of 15 or more cigarettes per day compared with 31% and 18% in Britain in 1976 (OPCS, 1978). (British data for current smokers are the average of data for 1980 and 1982, since 1981 data are not published; numbers of cigarettes smoked per day are for 1976, since published data are not suitably subdivided thereafter; ex-smoker data are for ex-cigarette smokers, averaging 1980 and 1982.) National data on tobacco consumption for the decades before the diagnosis of the cancers in this study (Beese, 1972) are less in accord with the lung cancer differences: with the exception of the period of the Second World War, tobacco consumption per adult was not greater in the UK than NZ. On the other hand, manufactured cigarette consumption was much greater in the UK than in NZ (Beese, 1972).

The low risk of lung cancer for New Zealanders in England and Wales would fit with the critical effect of early smoking in establishment of the smoking habit and risk of lung cancer (Peto, 1986). The intermediate risk for English and Welsh in NZ may in part reflect the establishment of English and Welsh smoking habits in this group at a young age, followed by a reduction in smoking after reaching NZ. The proportion of ex-smokers in British migrants to NZ (33% in males, 18% in females) in the 1981 NZ census (Department of Statistics, 1983) was greater than in either Britain (29% and 15% respectively; OPCS, 1984) or New Zealand non-Maoris in NZ (23% and 14%) in the same year, while the proportions of current smokers were similar in the British immigrants and New Zealand-born in NZ, and both were lower than in England and Wales. The intermediate risks may also reflect appreciable childhood migration, with adoption of NZ smoking habits by those who migrated young: about half of the English and Welsh in New Zealand in 1976 had migrated in or before their early twenties (Department of Statistics, unpublished data). Eastcott (1956) noted, more than 30 years ago, the raised risk of lung cancer in British migrants to NZ, particularly those who migrated at older ages, compared with NZ natives. He did not investigate whether the migrants' risk was lower than that in Britain.

Pleural cancer risks were far lower in NZ than in England and Wales, presumably because of lower levels of asbestos-related work in NZ.

Mean alcohol consumption per capita (in 1970–72) was appreciably greater in NZ than in the UK, and the prevalence of hepatitis B surface antigen (HbsAg) was also greater in NZ (Qiao *et al*., 1988). One might therefore expect a greater risk of primary liver cancer, for which these are the main known risk factors, in New Zealanders in NZ than in English and Welsh in England and Wales, and this was clearly the case in the weighted analyses at least. Male but not female migrants to NZ appear to have acquired these raised risks, again only clearly in the weighted analysis.

Among sites related to both alcohol and tobacco consumption, oropharyngeal and laryngeal cancers showed no consistent pattern. Oesophageal cancer risks in New Zealanders in NZ were below those in English and Welsh in England and Wales, and this reduction was shared by English and Welsh migrants to NZ, whereas NZ migrants to England and Wales of each sex had risks of oesophageal cancer 60% above those of English and Welsh natives, albeit based on small numbers. Haenszel (1982) has noted risks of oesophageal cancer above both host and native country rates in several groups of male migrants to the US, and suggested that this may be due to high alcohol consumption consequent on the stress of migration: the risks in New Zealanders in England and Wales, but not those in English and Welsh in NZ, would be compatible with this.

Stomach cancer risks were much lower in NZ than England and Wales, and significantly reduced risks compared with those in England and Wales (although above NZ-born levels) were present in English and Welsh migrants to NZ. Risks in New Zealanders in England and Wales were inconsistent, but based on fairly small numbers. Studies of migrants elsewhere have found a high risk of stomach cancer to be retained in migrants from high- to low-risk countries (Haenszel, 1982), suggesting a critical aetiological role for early life exposures. Since stomach cancer is a strongly social class-related malignancy, it is possible that if the migrants were selected by social class their risks might reflect social class-related behaviours, e.g. diet, before migration.

For pancreatic cancer, the low risk in New Zealanders in NZ was shared by the immigrants to NZ but not by New Zealanders in England and Wales. The approximate acquisition of host country risks by migrants would be compatible with aetiological factors acting late in life. Pancreatic cancer risks relate to smoking, and thus the low risk in New Zealanders in NZ accords with the lung cancer risks there. The pancreatic cancer risks in English and Welsh in NZ also accord to some extent with their lung cancer risk; the risks in New Zealanders in England and Wales do not, but since they are based on small numbers interpretation must be cautious.

Cervical cancer risks are complicated to assess since they reflect the extent of cervical screening and hysterectomy as well as the effects of aetiological factors for the malignancy. No data on these variables are available specifically for the migrants. Hysterectomy rates have been higher in New Zealand than in England and Wales (Macintosh, 1987), but this difference and plausible differences in screening rates could account for only a small part of the difference in risk. The lower ovarian cancer risks in New Zealanders than English and Welsh migrants in NZ, and in both groups than in English and Welsh in England and Wales, fit with their parities, since ovarian cancer risk is greatest with lowest parity. The risk for New Zealanders in England and Wales was not more than unity, as would be expected from their low parity, but unlike the risks in NZ there were wide confidence limits which included substantially raised risk.

Testicular cancer risks in NZ are among the highest in the world (Findlay *et al*., 1987). The raised risks also in English and Welsh migrants might indicate an effect of host country behaviours or environment on risk. It might also reflect their social class, however, if the migrants to NZ were of higher social class, as are those from NZ to England and Wales (we have no data on this for the migrants to NZ): testicular cancer is substantially more common in higher social classes.

Overall, cancers with sizeable significantly raised risk in both the weighted and unweighted analyses in each sex (or in one sex for sex-specific sites) seem the most likely to be of aetiological interest. Such raised risks were present in New Zealanders in NZ compared with English and Welsh in their home country for melanoma, cancers of the lip, mouth, small intestine, colon, thymus, prostate, eye and thyroid and non-Hodgkin's lymphoma. For each of these malignancies except mouth, small intestine and colon, there were also substantially raised risks in English and Welsh migrants to NZ, implying that the raised risk might be acquired by exposure to the NZ environment or behaviours later in life. For prostatic, thyroid and thymus cancers and non-Hodgkin's lymphoma, however, differences in diagnostic practices between NZ and England and Wales are an alternative explanation for the apparent differences in risk. For melanoma, colon and prostatic cancers and, based on small numbers, for lip cancer, there was raised risk in New Zealanders in England and Wales, implying that early NZ experiences or behaviours can alter risk. Cancers of the stomach, lung, pleura, cervix, ovary, scrotum and penis and bladder were at sizeable and significantly greater risk in English and Welsh in their home country than in New Zealanders in NZ without evident artefactual reasons for this. Of these tumours, for cancers of the stomach, cervix and ovary the risks in migrants may reflect selective characteristics of the migrants rather than the effects of their exposure to the environment or behaviours of the two countries, whereas the raised risks of lung, pleural, scrotal and penile and bladder cancers in English and Welsh migrants in NZ may reflect their exposures and behaviours before migration. In future it would be of interest to investigate how the risks of cancer in migrants between NZ and England and Wales vary with age at migration and duration since migration, particularly for colon cancer and melanoma.

**Acknowledgements**
We thank the Office of Population Censuses and Surveys for England and Wales data, and Mr J Fraser and Ms J Auld, National Health Statistics Centre, Wellington and Mr P Herbison, University of Otago, Dunedin for New Zealand data. The Epidemiological Monitoring Unit is funded by the Medical Research Council.

**References**

BEESE DH (ed.). (1972). *Tobacco Consumption in Various Countries*, Tobacco Research Council Research Paper 6, 3rd edn. Tobacco Research Council: London.

COOKE KR AND FRASER J. (1985). Migration and death from malignant melanoma. *Int. J. Cancer*, **36**, 175-178.

COX B AND LITTLE J. (1992). Reduced risk of colorectal cancer among recent generations in New Zealand. *Br. J. Cancer*, **66**, 386-390.

DEPARTMENT OF STATISTICS. (1980). *New Zealand Census of Population and Dwellings, 1976*, Vol. 7. *Birthplaces and Ethnic Origin*. Department of Statistics: Wellington.

DEPARTMENT OF STATISTICS. (1983). *New Zealand Census of Population and Dwellings 1981. Bulletin on Cigarette Smoking*. Department of Statistics: Wellington.

EASTCOTT DF. (1956). The epidemiology of lung cancer in New Zealand. *Lancet*, **i**, 37-39.

FERNS KA. (1974). Differential fertility among birthplace groups in New Zealand, Bsc(Hons) dissertation, Department of Geography, University of Otago, Dunedin.

FINDLAY FJ. (1989). *Cancer Data: New Registrations and Deaths 1984*. National Health Statistics Centre, Department of Health: Wellington.

FINDLAY FJ, FRASER J AND AULD JA. (1987). New Zealand. In *Cancer Incidence in Five Continents*, Vol. V. IARC Scientific Publication No 88. Muir C, Waterhouse J, Mack T, Powell J and Whelan S. (eds) pp. 754-765. IARC: Lyon.

HAENSZEL W. (1982). Migrant studies. In *Cancer Epidemiology and Prevention*. Schottenfeld D, Fraumeni Jr JF. (eds) W.B. Saunders: Philadelphia.

INTERNATIONAL AGENCY FOR RESEARCH ON CANCER. (1992). *IARC Monographs on the Evaluation of Carcinogenic risks to Humans*, Vol. 55. *Solar and Ultraviolet Radiation*. IARC: Lyon.

MACINTOSH MCM. (1987). Incidence of hysterectomy in New Zealand. *NZ Med. J.*, **100**, 345-347.

MACK TM AND FLODERUS B. (1991). Malignant melanoma risk by nativity, place of residence at diagnosis, and age at migration. *Cancer Causes Control*, **2**, 401-411.

MCKENZIE RL AND ELWOOD JM. (1990). Intensity of solar ultraviolet radiation and its implications for skin cancer. *NZ Med. J.*, **103**, 152-154.

MCLINTOCK AH. (1966). Immigration. In *An Encyclopedia of New Zealand*. Vol. 2, pp. 130-139. Government Printer: Wellington.

MANTEL N AND HAENSZEL W. (1959). Statistical aspects of the analysis of data from retrospective studies of disease. *J. Natl Cancer Inst.*, **22**, 719-748.

MUIR C, WATERHOUSE J, MACK T, POWELL J AND WHELAN S (eds). (1987). *Cancer Incidence in Five Continents*, Vol. V. IARC Scientific Publication No. 88. IARC: Lyon.

OFFICE OF POPULATION CENSUSES AND SURVEYS, SOCIAL SURVEY DIVISION. (1978). *The General Household Survey 1976*. HMSO: London.

OFFICE OF POPULATION CENSUSES AND SURVEYS, SOCIAL SURVEY DIVISION. (1984). *The General Household Survey 1982*. HMSO: London.

PETO R. (1986). Influence of dose and duration of smoking on lung cancer rates. In *Tobacco: A Major International Health Hazard*, IARC Scientific Publication No. 74, Zaridze DG and Peto R. (eds) pp. 23-33. IARC: Lyon.

QIAO Z-K, HALLIDAY ML, RANKIN JG AND COATES RA. (1988). Relationship between hepatitis B surface antigen prevalence, per capita alcohol consumption and primary liver cancer death rate in 30 countries. *J. Clin. Epidemiol.*, **41**, 787-792.

SNOVER DC, LEVINE GD AND ROSAI J. (1982). Thymic carcinoma. Five distinct histological variants. *Am. J. Surg. Pathol.*, **6**, 451-470.

SWERDLOW AJ. (1986). Cancer registration in England and Wales: some aspects relevant to interpretation of the data. *J. R. Stat. Soc. A*, **149**, 146-160.

SWERDLOW AJ, DOUGLAS AJ, VAUGHAN HUDSON G AND VAUGHAN HUDSON B. (1993). Completeness of cancer registration in England and Wales: an assessment based on 2,145 patients with Hodgkin's disease independently registered by the British National Lymphoma Investigation. *Br. J. Cancer*, **67**, 326-329.

SWERDLOW AJ AND DOS SANTOS SILVA I. (1991). Geographic distribution of lung and stomach cancers in England and Wales over 50 years: changing and unchanging patterns. *Br. J. Cancer*, **63**, 773-781.

WORLD HEALTH ORGANIZATION. (1967). *Manual of the International Statistical Classification of Diseases, Injuries, and Causes of Death*, 8th revision. WHO: Geneva.

WORLD HEALTH ORGANIZATION. (1977). *Manual of the International Statistical Classification of Diseases, Injuries, and Causes of Death*, 9th revision. WHO: Geneva.